# Mini-review on human-centered assurance in robot-assisted orthopedics and neurosurgery

**DOI:** 10.3389/frobt.2026.1755883

**Published:** 2026-02-23

**Authors:** Sue Min Cho, Xinrui Zou, Laura Fleig, Mathias Unberath

**Affiliations:** Department of Computer Science, Johns Hopkins University, Baltimore, MD, United States

**Keywords:** autonomous systems, human-robot interaction, medical robotics, safety validation, surgical automation

## Abstract

As artificial intelligence (AI) drives the development of next-generation robotic platforms and navigation systems that operate with increasing levels of autonomy in orthopedic and neurosurgical procedures, the methods by which human operators verify and validate these systems’ operations become critically important. While significant effort has been spent on advancing technological capabilities and autonomy, comparatively little thought has been put into understanding how surgeons may effectively maintain oversight and assurance of these complex systems–despite retaining full legal and ethical responsibility for surgical outcomes. This mini-review synthesizes assurance mechanisms following the Sense-Think-Act framework: spatial intelligence (navigation and registration), cognitive assistance (AI-driven planning and adaptation), and physical operation (robot motion and force interaction). We highlight human-centered assurance as an opportunity to enable safe adoption of increasingly autonomous surgical systems. Finally, we outline essential research directions for developing assurance frameworks that scale with increasing autonomy while maintaining human responsibility and control in orthopedic and neurosurgical procedures.

## 1 Introduction

The integration of robot-assisted surgical systems into orthopedic and neurosurgical procedures has revolutionized surgical practice, offering unprecedented precision and consistency for challenging minimally invasive approaches (Osman et al., 2025; Bunch et al., 2025; Ram et al., 2023; Doulgeris et al., 2015). These specialties, where millimeter errors can be catastrophic and actions like bone removal are irreversible, particularly benefit from robotic assistance. However, as these systems grow more sophisticated–incorporating increase in autonomy, advanced navigation capabilities, and AI-driven decision support–a critical challenge emerges: ensuring that human operators can effectively verify, validate, and maintain oversight of their integrated operation (Yang et al., 2017).

Despite substantial efforts devoted to advancing technological capabilities, the methods by which human operators assure safe and correct system operation remain comparatively underexplored. This gap is particularly concerning given that legal and ethical responsibility for surgical outcomes remains firmly with the humans, regardless of the level of technological assistance employed (Fosch-Villaronga et al., 2021; O’Sullivan et al., 2019). While a robotic system may execute a bone cut or an AI algorithm may suggest an optimal trajectory, the surgeon must ultimately ensure these actions are appropriate, accurate, and safe for each individual patient.

Given the complex and variable nature of surgical interventions, including anatomical variation, unforeseen complications, and dynamic tissue interactions, surgical systems must be designed to preserve meaningful human oversight. This human-centered assurance, which we define as the methods and interfaces enabling surgeons to monitor, verify, and intervene in robot-assisted surgical systems, represents a critical opportunity to enable the safe adoption of increasingly autonomous surgical technologies. Lessons from autonomous vehicle development demonstrate that effective human-machine teaming requires deliberate design of trust calibration mechanisms, graduated autonomy frameworks, and transparent system communication (Lee and See, 2004; Parasuraman and Manzey, 2010; Committee, 2021). Similarly, regulatory bodies, including the FDA, have begun requiring transparency and human oversight provisions for AI-enabled medical devices (Shah et al., 2025), recognizing that meaningful human control is foundational to safe deployment.

This evolving landscape presents a significant opportunity: proactively developing human-centered assurance methods that scale alongside technological advancement. Rather than viewing oversight as a constraint on autonomy, effective assurance frameworks can enable the safe deployment of more capable systems by ensuring that human operators maintain calibrated trust and appropriate situational awareness (Endsley, 2017; Hoff and Bashir, 2015). The autonomous driving domain illustrates this principle through the SAE levels of driving automation, which explicitly define human roles and responsibilities at each autonomy level (Committee, 2021). A similar framework for surgical robotics could facilitate systematic development of assurance methods matched to system capabilities.

This opportunity manifests across three interconnected components that mirror the canonical robotics pipeline of Sense-Think-Act. First, spatial intelligence (Sense) integrates multiple imaging and tracking modalities to create the perceptual foundation that guides all subsequent operations. Effective assurance methods can help surgeons maintain the perceptual grounding needed to validate this spatial understanding. Second, cognitive assistance (Think) leverages AI to analyze surgical data and recommend optimal approaches. Transparent, explainable systems can enable surgeons to appropriately calibrate trust in these recommendations. Third, physical operation (Act) translates plans into robot movements and force interactions. Well-designed feedback mechanisms can ensure surgeons maintain meaningful oversight of autonomous execution. This Sense-Think-Act framework not only reflects the logical flow of autonomous systems but also provides a natural structure for developing corresponding assurance methods.

This mini-review provides a focused exploration of human-centered assurance methods in robotic-assisted orthopedic and neurosurgical procedures, organized according to the Sense-Think-Act framework: 1. assuring spatial intelligence (Sense); 2. assuring cognitive assistance (Think); and 3. assuring physical operation (Act). Table 1 provides an overview of the assurance methods discussed in the following sections, organized according to this framework.

**TABLE 1:** Current human-centered assurance methods in robot-assisted orthopedic and neurosurgery.

Framework component	Assurance method	Key approaches
Spatial intelligence (sense)	Tracking technologies	Optical tracking, electromagnetic tracking, marker-based registration
	Image-based navigation	Fluoroscopy, CT, optical cameras, ultrasound-guided registration
	AR/MR visualization	Reflective-AR displays, markerless AR alignment verification
	Uncertainty communication	Registration confidence metrics, spatially-varying confidence visualization
Cognitive assistance (think)	Explainable AI	Saliency maps, interpretable model design, decision transparency
	Uncertainty quantification	Prediction reliability indicators, confidence-aware recommendations
	Agentic systems	Language-guided control, human-interpretable action representations
Physical operation (act)	Shared control	Virtual boundaries, motion constraints, tremor suppression
	Multimodal feedback	Haptic feedback, visual-attention modeling, AR trajectory visualization
	Confidence-based control	Dynamic autonomy adjustment, uncertainty-aware execution
	Predictive simulation	Digital twins, pre-execution movement preview

Through this analysis, we aim to synthesize current approaches, identify opportunities for advancement, and outline essential directions for future research. Our goal is to inform the development of assurance frameworks that empower humans to maintain meaningful oversight as orthopedic and neurosurgical technologies continue to advance, ultimately ensuring that these advanced systems enhance rather than compromise surgical safety and effectiveness.

## 2 Assuring spatial intelligence: navigation and registration quality

Spatial intelligence, the system’s understanding of where surgical instruments are relative to patient anatomy, forms the perceptual foundation (Sense) upon which all subsequent cognitive planning and physical execution depend. Before a robotic system can recommend an optimal trajectory or execute a precise movement, it must first establish and maintain accurate spatial alignment between its coordinate system, surgical tools, and patient anatomy. Assuring this spatial understanding is therefore foundational: errors at the sensing stage propagate through and corrupt all downstream operations, regardless of how sophisticated the planning algorithms or how precise the mechanical execution (Gundle et al., 2017; Langlotz, 2004; Haidegger et al., 2009).

Current robotic systems rely on various tracking technologies (e.g., optical, electromagnetic) to establish and maintain spatial alignment (Saeedi-Hosseiny et al., 2023; Aguilera Saiz et al., 2024). While these conventional approaches offer the advantage of clear failure modes, they require invasive marker placement and external equipment (Šuligoj et al., 2017; Kord et al., 2021).

Image-based navigation promises to overcome these limitations through markerless registration and reduced setup complexity by leveraging imaging equipment already present in most operating rooms. Fluoroscopy-based approaches are common in orthopedic and neurosurgical settings, and RGB-D cameras can also enable markerless registration (Liebmann et al., 2024). Machine learning methods have further begun to address registration challenges (Unberath et al., 2021). Yet even sophisticated systems commonly produce registration errors exceeding clinically acceptable thresholds.

While human surgeons recognize and compensate for subtle misalignments through experience and contextual awareness, current robotic systems typically execute plans without independent verification of registration validity, meaning registration errors directly translate into misplaced instruments or implants unless explicitly detected by the system or operator.

Early attempts at automated registration verification have shown limited success. Varnavas et al. (2015) developed automated 2D/3D registration verification but it is constrained by restrictive assumptions that fail to generalize and even within their specific application, the method failed in over 6% of cases, highlighting the inadequacy of purely algorithmic approaches for this safety-critical task. More recent learning-based registration systems, including neural rendering approaches for cross-modal 2D/3D alignment (Fehrentz et al., 2024), demonstrate improved registration performance under challenging visual conditions. Like many prior registration methods, they are primarily evaluated in terms of alignment accuracy, with less emphasis on how operators can assess or verify correctness during use.

Recognizing such limitations, researchers have shifted toward human-in-the-loop verification methods. For 3D-to-3D alignment in augmented reality (AR) and mixed reality (MR) scenarios, progress has been notable. Fotouhi et al. (2020) demonstrated that reflective-AR displays improve alignment precision by helping surgeons better align virtual and physical environments. Similarly, Kantak et al. (2024) showed that markerless AR systems could improve targeting accuracy of skull landmarks. These successes in 3D-to-3D scenarios highlight the potential of AR for spatial verification.

However, 2D-to-3D registration–also critical for image-guided robotic navigation–poses distinct challenges that remain largely unresolved. Our previous work (Cho et al., 2023) explored visualization paradigms for assessing 2D/3D registration in robotic spine surgery, finding that while users could differentiate error magnitudes better than chance, performance remained insufficient for reliable verification. Similar findings in pelvic registration (Cho et al., 2025b) confirm that visualization techniques alone cannot ensure robust verification.

Researchers have also explored communicating registration uncertainty to enable more nuanced assurance of spatial alignment quality. In prior work, we (Cho et al., 2025a) quantified the relationship between registration uncertainty metrics and actual accuracy, which can potentially provide operators with confidence indicators. Geshvadi et al. (2025) developed methods to visualize how registration confidence varies across the surgical field during virtual tumor resection. This approach enables surgeons to understand not just whether registration is accurate globally, but where it can be trusted.

Finally, recent work suggests that spatial intelligence in surgery extends well beyond estimating a single camera, tool, or anatomy alignment. Multimodal datasets such as MM-OR model the operating room as a dynamic scene of staff, tools, robots, and equipment using synchronized RGB-D, audio, robotic logs, and semantic scene graphs (Özsoy et al., 2025). This perspective shows that spatial understanding is a multimodal, relational problem that requires reliable ways to assure spatial intelligence in complex surgical settings.

## 3 Assuring cognitive assistance: AI-driven planning and adaptation

Once spatial understanding is established, the cognitive assistance component (Think) analyzes this information alongside clinical data to generate surgical recommendations. Modern robotic surgical systems increasingly incorporate AI components for preoperative planning, intraoperative trajectory optimization, and real-time adaptation to surgical conditions. This cognitive layer transforms robots from precise positioning devices into intelligent surgical assistants capable of analyzing imaging data, suggesting optimal approaches, and adapting plans based on intraoperative findings. The opacity of these AI-driven decisions creates distinct assurance challenges, requiring methods that enable surgeons to interpret, validate, and appropriately trust algorithmic recommendations.

As such, the integration of AI introduces promising developments in orthopedic and neurosurgical robotics, but challenges persist, including data heterogeneity, algorithmic bias, and the “black box” nature of many models, alongside issues with robust validation (Misir and Yuce, 2025). These challenges are particularly acute in robotic surgery, where AI recommendations can directly translate into physical robotic actions with immediate patient impact, and the opacity of these AI-driven decisions creates unique assurance challenges that differ from mechanical or spatial verification.

Recognition of these limitations has sparked initial efforts toward AI transparency in surgical contexts. These efforts align with research in explainable artificial intelligence (XAI) for robotics, which seeks to make perception, planning, and control decisions interpretable to human operators, particularly in safety-critical settings (Anjomshoae et al., 2019). Within surgical contexts, Han et al. (2025) articulates the critical need for AI systems with built-in explainability to enable clinicians to interpret and challenge model decisions. Tafti et al. (2025) demonstrates how uncertainty quantification can highlight when predictions are unreliable, potentially prompting manual verification. However, these remain largely conceptual frameworks rather than implemented solutions in current robotic systems.

Some promising approaches are beginning to emerge from adjacent fields. In diagnostic imaging, saliency maps highlight which image regions influenced AI decisions, a technique that could be adapted to show why certain trajectories were selected. Amirian et al. (2023) identify opportunities for such explainable AI in orthopedics, though they acknowledge the tradeoff between interpretability and model performance.

In parallel, emerging work on agentic systems and language-guided control from the broader robotics community offers a complementary perspective on explainability by emphasizing structured and human-interpretable representations of action. Rather than relying solely on *post hoc* explanations, these approaches express planning and adaptation through explicit decision steps that can be examined, queried, or overridden by human operators. Prior work on explainable agents and robotic decision-making suggests that grounding actions in interpretable intermediate representations, including natural language, can support transparency and accountability in safety-critical settings (Anjomshoae et al., 2019; Huang et al., 2022). Recent work has demonstrated the feasibility of language-guided control in surgical settings, enabling natural language commands to control robotic X-ray systems (Killeen et al., 2024) and leveraging language-promptable digital twins for intelligent device control (Killeen et al., 2025). Beyond device control, language-based interfaces have also been explored for intraoperative surgical assistance, moving toward more natural human-machine collaboration during procedures (Seenivasan et al., 2025). Although fully agentic paradigms remain largely unexplored in current orthopedic and neurosurgical robotic systems, these developments highlight a promising direction for aligning AI-driven planning with the assurance requirements of intraoperative workflows.

The regulatory landscape is beginning to drive progress. Panesar et al. (2020) note that some regulators now mandate algorithmic transparency before approving AI systems for patient care, creating pressure for more interpretable systems. Shahid et al. (2025) found that lack of transparency remains the primary barrier to AI adoption in spinal and cranial surgery, suggesting that even basic explainability features could significantly impact clinical acceptance and safe integration with robotic systems.

Despite these developments, current robotic surgical systems with AI components largely maintain clear separation between AI recommendations and robotic execution. The surgeon must actively transfer AI suggestions into robot commands, preserving human oversight but limiting seamless integration. Future systems will need to develop real-time explainability suited to surgical workflows, confidence-aware execution that automatically adjusts robot autonomy based on AI certainty, and clear frameworks for which decisions require human validation versus autonomous execution.

## 4 Assuring physical operation: movement and mechanical safety

Building upon spatial understanding (Sense) and cognitive plans (Think), the physical operation component (Act) represents where robotic systems directly interact with patient anatomy. While errors in navigation or AI systems manifest initially as incorrect information that may propagate to cause harm, errors during physical execution result in immediate patient impact at the point of contact. This distinction introduces the unique assurance challenges of the execution phase: even when spatial registration is accurate and AI recommendations are appropriate, the physical robot must faithfully translate these inputs into safe movements and force interactions. Errors in robotic execution can cause immediate physical harm through unintended movements, excessive forces, or spatial inaccuracies (Alemzadeh et al., 2016; Agcaoglu et al., 2012; Rivero-Moreno et al., 2023; Pagani et al., 2022). In neurosurgical and orthopedic procedures, where surgeons work near critical structures and rely on precise bone preparation, even millimeter-scale position errors or modest force overshoots can result in nerve damage, vascular injury, or compromised implant fixation (Gurses et al., 2024; Moccia et al., 2018; Karas and Chiocca, 2007; Lee et al., 2024; Mulyadi et al., 2024).

Most current robotic surgical systems keep the surgeon in the loop, with the robot providing assistance such as tremor suppression, precise positioning, or motion constraints (Rivero-Moreno et al., 2023; Abdelaal et al., 2020; Tomasz et al., 2021). Grounded shared control can constrain tools away from anatomically critical regions, provided accurate co-registration between the robot frame and the patient anatomy (Payne et al., 2020). These preprogrammed constraints create virtual boundaries that prevent both the surgeon and the robot from entering dangerous zones.

However, as autonomy increases, robotic systems will independently execute portions of the procedure with the surgeon supervising and intervening when needed (Liu et al., 2024). This fundamental shift towards supervisory oversight requires new capabilities: surgeons must maintain situational awareness, be able to rapidly assess system confidence and performance, and access intervention mechanisms that are both responsive and minimally disruptive to workflow.

Similar challenges have already emerged in other human-robot interaction domains, including collaborative industrial robots, mobile service robots, and semi-autonomous vehicles. In these settings, humans no longer continuously control motion but instead supervise automated behaviors, intervening only when necessary (Endsley, 2017). To support this mode of interaction, these fields have developed methods for intent prediction, shared control, confidence-aware autonomy, and transparent system feedback. For example, cobots use adaptive impedance control, safety envelopes, and real-time human motion prediction to safely operate in close proximity to workers (Haddadin et al., 2017; Mainprice and Berenson, 2013; Lasota et al., 2017), while autonomous driving systems rely on uncertainty-aware planning, driver monitoring, and graded autonomy handoff mechanisms to maintain human readiness (Doshi and Trivedi, 2011; Fridman, 2018). Across these domains, effective assurance depends on both low-level safety constraints and higher-level mechanisms that communicate system intent, limitations, and confidence to human supervisors. These strategies suggest that surgical robotics can draw from a broader body of work on supervisory control, human trust calibration, and situation-aware autonomy when designing assurance mechanisms for physical operation.

Recognizing this gap, several emerging technologies show promise for potential physical operation assurance in robotic surgery. Digital twin technology (Ding et al., 2024; Kyeremeh et al., 2025) and simulation approaches (Killeen et al., 2023) can create high-fidelity virtual replicas of robotic systems and patient anatomy, potentially enabling surgeons to preview robotic actions before execution and maintain parallel virtual monitoring during procedures. This technology could provide predictive assurance by simulating planned movements and their potential consequences before physical execution.

AR and MR technologies represent another promising avenue, demonstrating how visualization can enhance oversight capabilities. Vörös et al. (2022) developed an AR-based interaction scheme for robotic pedicle screw placement that not only visualizes planned trajectories but also enables intraoperative plan adjustment and direct robot control. This bidirectional interaction represents a significant advance in maintaining human oversight during robotic procedures. The potential of combining multiple sensory modalities for assurance is also shown in recent work by Chen et al. (2024), who demonstrated that integrating AR visualization with haptic feedback and real-time visual-attention modeling creates a safe and ergonomic shared-control framework for robot-assisted pedicle screw drilling. Their system outperformed both full human and full robot control, suggesting that effective assurance may require multimodal feedback that aligns with surgeons’ natural perception-action coupling.

Beyond visualization and haptic feedback, communicating robot uncertainty and confidence levels represents a critical yet underdeveloped aspect of physical operation assurance. While not specific to orthopedic or neurosurgical applications, work in soft tissue surgery provides valuable insights that could be adapted. Saeidi et al. (2018) developed a confidence-based shared control strategy for the Smart Tissue Autonomous Robot (STAR) that reduced operator work time compared to pure manual control while maintaining safety through dynamic automation adjustment based on system confidence. Similarly, Kam et al. (2021) demonstrated confidence-based supervised-autonomous control for robotic vaginal cuff closure, showing how robots can communicate their certainty levels to guide appropriate human oversight.

In neurosurgical contexts, uncertainty quantification takes on additional complexity due to tissue deformation and the critical nature of anatomical structures. Frisken et al. (2021) advanced this field by moving beyond simple safety margins to incorporate multiple uncertainty sources. Their approach combines segmentation uncertainty and predicted brain shift into unified risk volumes, demonstrating how comprehensive uncertainty quantification can inform safer robotic operation. This multi-factorial approach to uncertainty could be extended to real-time applications, providing surgeons with dynamic confidence information throughout procedures. Extending this assurance to the physical workspace, Xian et al. (2025) employed CLF-CBF constraints to guarantee the safety of the supervising surgeon during close-range human-robot interaction.

## 5 Discussion

Our analysis, organized around the Sense-Think-Act framework, reveals significant opportunities to advance human-centered assurance in robot-assisted orthopedic and neurosurgical procedures. While current efforts remain fragmented, promising foundations have been established across all three components of robotic systems. For physical operation, shared control paradigms and multimodal feedback demonstrate potential for maintaining effective human oversight. For spatial intelligence, innovative visualization methods are beginning to help surgeons detect registration errors, though reliability improvements are needed. For AI assistance, growing recognition of explainability needs is driving initial development efforts, even as implementation remains in early stages.

As robotic systems become more sophisticated and gain autonomy, the need for effective human oversight becomes paradoxically more critical yet more challenging. This evolution presents opportunities for transformative advances in several key areas. First, integrated assurance architectures can move beyond separate solutions for physical, spatial, and cognitive components toward unified frameworks that propagate uncertainty and confidence information across all system aspects. When registration quality degrades, for instance, this information should automatically influence both physical execution parameters and the confidence in AI decisions. Second, predictive methods can enable operators to anticipate potential failures before they occur, shifting from reactive error detection to proactive risk management. Third, context-aware adaptation can address how assurance requirements vary dramatically by anatomical location and procedure phase, with systems dynamically adjusting oversight sensitivity based on clinical context.

The transition from human-operated to human-supervised robotic surgery represents a fundamental shift that current assurance methods are approaching but have not yet fully addressed. As autonomy increases, the surgeon’s role evolves from direct controller to system supervisor–a transformation requiring new interfaces, training paradigms, and conceptual frameworks for human-robot collaboration. Critical to this evolution is validated human factors design through extensive user studies. As emphasized in our prior work (Cho et al., 2025b), ecological validity requires testing under realistic surgical conditions, as laboratory findings may not translate to the operating room, where the perceived stakes modulate participant perception and behavior. Future systems must therefore be developed and validated in environments that capture the full complexity of surgical practice, ensuring that assurance methods work not just in principle but in the demanding reality of orthopedic and neurosurgical procedures.

## 6 Conclusion

The future of robot-assisted orthopedic and neurosurgery depends on both advancing technical capabilities and ensuring that these systems remain transparent, predictable, and meaningfully controllable to human operators. As surgical technologies continue to evolve in complexity and capability, parallel innovation in human-centered assurance methods is foundational to enable effective human-machine collaboration in safety-critical environments. Realizing this goal requires a shift from the current paradigm of humans adapting to technology to one in which technology is deliberately designed to augment human roles and responsibilities. Only through such proactive, human-centered approaches can emerging surgical technologies fulfill their promise of excellent surgical care while maintaining the safety, accountability, and trust.
